# Supplementary material for: Cooperative Conformational Transitions Underpin the Activation Heat Capacity in the Temperature Dependence of Enzyme Catalysis
Source: ACS Catal. 2024 Mar 8;14(7):4379–94. doi: 10.1021/acscatal.3c05584 (PMC11020164; doi:10.1021/acscatal.3c05584)
Supplement: Supplementary file 1 — cs3c05584_si_001.pdf [file cs3c05584_si_001.pdf]

Supporting Information

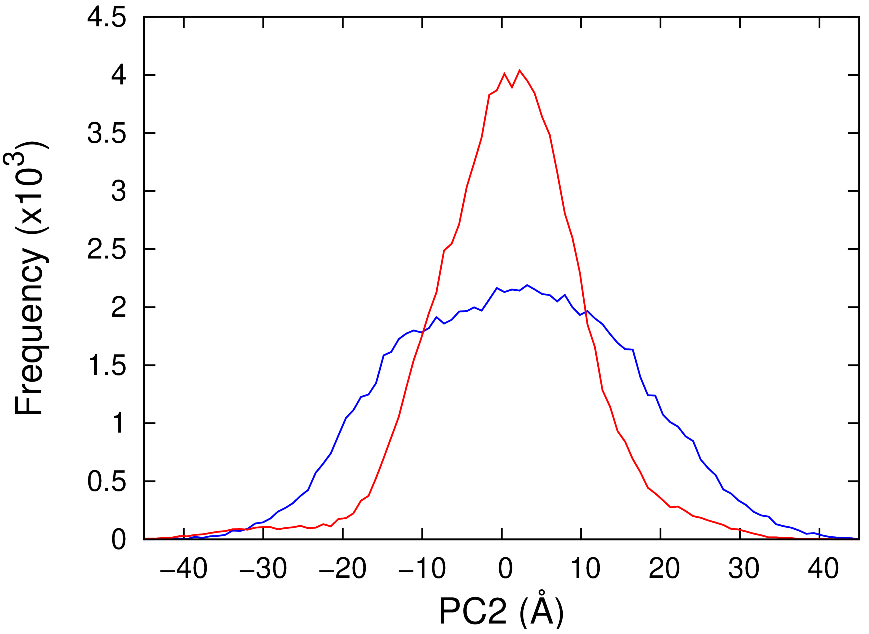

Figure S1. Principal component analysis for WT MalL in the ES (blue) and E-TS (red) states showing a projection of the second principal component, PC2.

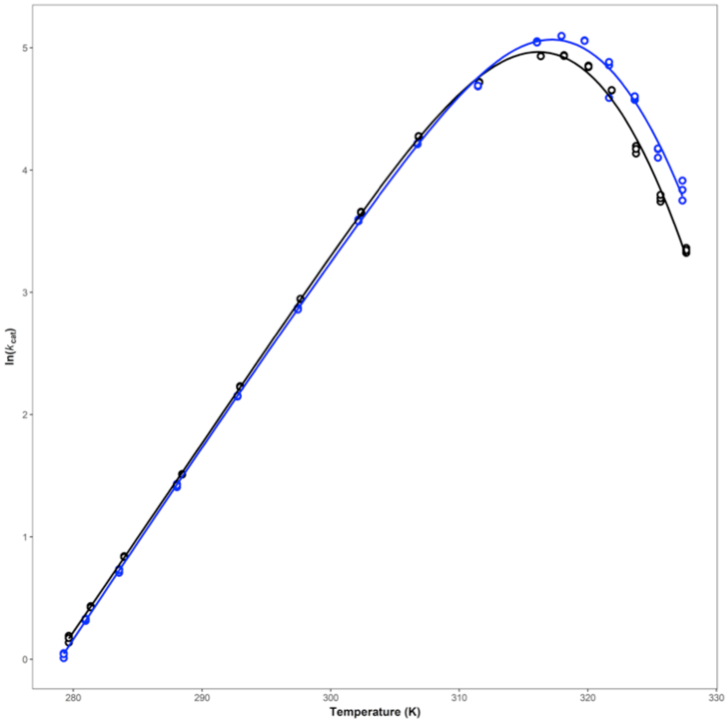

Figure S2. Ln(rate) versus temperature for WT MalL (black) and S536R (blue).

Table S1. Data collection and refinement statistics for MalL S536R.

Statistic	MalL S536R
Wavelength (Å)	0.953735
Space group	$P\ 1\ 2_1\ 1$
Unit cell lengths (Å)	a = 48.75 b = 101.00 c = 61.75
Unit cell angles (°)	α = 90.00 β = 113.06 γ = 90.00
Resolution (Å)	44.85 - 1.10 (1.12 - 1.10)
R_{merge}	0.107 (0.548)
Completeness (%)	94.2 (87.1)
Redundancy	10.9 (6.9)
No. of observations	2270037 (65265)
No. of unique reflections	208774 (9509)
Mean I/σI	12.7 (2.9)
R factor	0.126
R_{free}	0.145
Protein atoms	9633
Solvent atoms	785
Average temperature factor (Å^2)	16.13
RMSD bond lengths (°)	0.01
RMSD bond angles (Å)	1.182
